# Bis(μ-biphenyl-2,2′-dicarboxyl­ato)bis­[aqua­(4,4′-dimethyl-2,2′-bipyridine-κ^2^ *N*,*N*′)copper(II)]

**DOI:** 10.1107/S1600536809040628

**Published:** 2009-10-17

**Authors:** Xi-Yan Dong, Xiao-Jie Xu, Lei Yang

**Affiliations:** a Department of Physics and Chemistry, Henan Polytechnic University, Jiaozuo 454000, Henan, People’s Republic of China

## Abstract

The mol­ecule of the title binuclear copper(II) complex, [Cu_2_(C_14_H_8_O_4_)_2_(C_12_H_12_N_2_)_2_(H_2_O)_2_], is bis­ected by a crystallographic twofold axis. Each Cu^II^ atom is coordinated in a distorted octa­hedral geometry by three O atoms from two biphen­yl-2,2′-dicarboxyl­ate anions, one aqua O atom and two N atoms of a 4,4′-dimethyl-2,2′-bipyridine ligand. Intramolecular O—H⋯O hydrogen bonds between the coordinated water molecules and the carboxylate O atoms are also present.

## Related literature

For related structures, see: Li *et al.* (2009 ▶); Jiang & Feng (2009 ▶); Xu *et al.* (2009 ▶); Zhang *et al.* (2009 ▶); Rizal & Ng (2009 ▶); Zhang (2009 ▶).
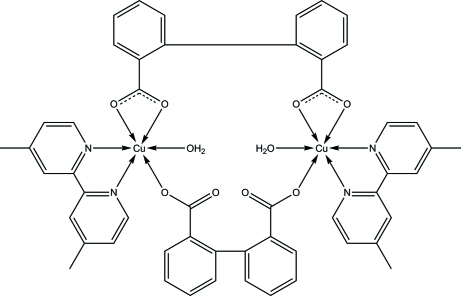


## Experimental

### 

#### Crystal data

[Cu_2_(C_14_H_8_O_4_)_2_(C_12_H_12_N_2_)_2_(H_2_O)_2_]
*M* *_r_* = 1109.71
Monoclinic, 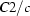

*a* = 17.104 (3) Å
*b* = 15.395 (2) Å
*c* = 18.289 (3) Å
β = 104.413 (3)°
*V* = 4664.2 (13) Å^3^
*Z* = 4
Mo *K*α radiation
μ = 0.98 mm^−1^
*T* = 296 K
0.26 × 0.24 × 0.22 mm

#### Data collection

Bruker SMART APEXII CCD area-detector diffractometer
Absorption correction: multi-scan (*SADABS*; Bruker, 2005 ▶) *T*_min_ = 0.785, *T*_max_ = 0.814
13512 measured reflections
4594 independent reflections
3108 reflections with *I* > 2σ(*I*)
*R*_int_ = 0.060

#### Refinement

*R*[*F* ^2^ > 2σ(*F* ^2^)] = 0.047
*wR*(*F* ^2^) = 0.109
*S* = 0.93
4594 reflections
310 parameters
12 restraints
H-atom parameters constrained
Δρ_max_ = 0.87 e Å^−3^
Δρ_min_ = −0.33 e Å^−3^

### 

Data collection: *APEX2* (Bruker, 2005 ▶); cell refinement: *SAINT* (Bruker, 2005 ▶); data reduction: *SAINT*; program(s) used to solve structure: *SHELXS97* (Sheldrick, 2008 ▶); program(s) used to refine structure: *SHELXL97* (Sheldrick, 2008 ▶); molecular graphics: *SHELXTL* (Sheldrick, 2008 ▶); software used to prepare material for publication: *SHELXTL*.

## Supplementary Material

Crystal structure: contains datablocks I, global. DOI: 10.1107/S1600536809040628/jh2107sup1.cif

Structure factors: contains datablocks I. DOI: 10.1107/S1600536809040628/jh2107Isup2.hkl

Additional supplementary materials:  crystallographic information; 3D view; checkCIF report

## Figures and Tables

**Table 1:** Hydrogen-bond geometry (Å, °)

*D*—H⋯*A*	*D*—H	H⋯*A*	*D*⋯*A*	*D*—H⋯*A*
O1*W*—H1*WB*⋯O4^i^	0.85	1.78	2.632 (4)	174
O1*W*—H1*WA*⋯O1^i^	0.85	1.95	2.782 (4)	164

Symmetry code: (i) 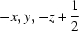.

**Fig. 1.**
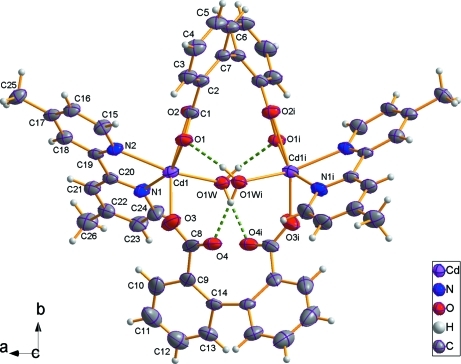
The molecular structure of the title compound with the atom-labelling scheme. Displacement ellipsoids are drawn at the 30% probability level. H atoms are presented as small spheres of arbitrary radius. Hydrogen-bond interactions are drawn with dashed lines. Atoms labeled(i) are generated by the symmetry code -x, y, -z + 1/2.

## supplementary crystallographic information

### Comment

The title binuclear copper(II) complex, [Cu_2_(C_14_H_8_O_4_)_2_(C_10_H_8_N_2_)_2_(H_2_O)_2_], is a centrosymmetric dimer. The asymmetric unit consitsts of one Cu^II^ atom, one 4,4'-dimethyl-2,2'-bipyridine (dbpy) ligand, one [1,1'-biphenyl]-2,2'-dicarboxylate dianion (bpdc^2-^) and a coordinated water molecule.

The Cu^II^ atom is six-coordinated by two N atoms from bpy and four O atoms, three from two bpdc^2-^ anions and one from coordinated H_2_O, in a distorted octahedron coordination geometry. And it is noteworthy that the two Cu^II^ ions in the complex are bridged by two bpdc^2-^ dianions, one is in a bis-monodentate mode whereas the other is in a bis-bidentate mode.

### Experimental

The title complound was synthesized hydrothermally in a Teflon-lined autoclave (25 ml) by heating a mixture of H_2_bpdc (0.2 mmol), dbpy (0.4 mmol) and CuSO_4_.5H_2_O (0.2 mmol) in water (10 ml) at 393 K for 3 d. Crystals suitable for X-ray analysis were obtained.

### Refinement

All H atoms were included in calculated positions, with C—H bond lengths fixed at 0.96 Å (methyl CH_3_), 0.93Å (aryl group) and O—H = 0.85 Å and were refined in the riding-model approximation. *U*_iso_(H) values were calculated at 1.5 *U*_eq_(C) for methyl groups and 1.2 *U*_eq_(C) otherwise.

### Crystal data

**Table:**

[Cu_2_(C_14_H_8_O_4_)_2_(C_12_H_12_N_2_)_2_(H_2_O)_2_]	*F*(000) = 2240
*M**_r_* = 1109.71	*D*_x_ = 1.580 Mg m^−^^3^
Monoclinic, *C*2/*c*	Mo *K*α radiation, λ = 0.71073 Å
Hall symbol: -C 2yc	Cell parameters from 3688 reflections
*a* = 17.104 (3) Å	θ = 2.7–27.3°
*b* = 15.395 (2) Å	µ = 0.98 mm^−^^1^
*c* = 18.289 (3) Å	*T* = 296 K
β = 104.413 (3)°	Block, colourless
*V* = 4664.2 (13) Å^3^	0.26 × 0.24 × 0.22 mm
*Z* = 4	

### Data collection

**Table:**

Bruker SMART APEXII CCD area-detector diffractometer	4594 independent reflections
Radiation source: fine-focus sealed tube	3108 reflections with *I* > 2σ(*I*)
graphite	*R*_int_ = 0.060
φ and ω scans	θ_max_ = 26.0°, θ_min_ = 1.8°
Absorption correction: multi-scan (*SADABS*; Bruker, 2005)	*h* = −21→20
*T*_min_ = 0.785, *T*_max_ = 0.814	*k* = −18→12
13512 measured reflections	*l* = −22→22

### Refinement

**Table:**

Refinement on *F*^2^	Primary atom site location: structure-invariant direct methods
Least-squares matrix: full	Secondary atom site location: difference Fourier map
*R*[*F*^2^ > 2σ(*F*^2^)] = 0.047	Hydrogen site location: inferred from neighbouring sites
*wR*(*F*^2^) = 0.109	H-atom parameters constrained
*S* = 0.93	*w* = 1/[σ^2^(*F*_o_^2^) + (0.0562*P*)^2^] where *P* = (*F*_o_^2^ + 2*F*_c_^2^)/3
4594 reflections	(Δ/σ)_max_ = 0.001
310 parameters	Δρ_max_ = 0.87 e Å^−^^3^
12 restraints	Δρ_min_ = −0.33 e Å^−^^3^

### Special details

**Table:**

Geometry. All e.s.d.'s (except the e.s.d. in the dihedral angle between two l.s. planes) are estimated using the full covariance matrix. The cell e.s.d.'s are taken into account individually in the estimation of e.s.d.'s in distances, angles and torsion angles; correlations between e.s.d.'s in cell parameters are only used when they are defined by crystal symmetry. An approximate (isotropic) treatment of cell e.s.d.'s is used for estimating e.s.d.'s involving l.s. planes.
Refinement. Refinement of *F*^2^ against ALL reflections. The weighted *R*-factor *wR* and goodness of fit *S* are based on *F*^2^, conventional *R*-factors *R* are based on *F*, with *F* set to zero for negative *F*^2^. The threshold expression of *F*^2^ > σ(*F*^2^) is used only for calculating *R*-factors(gt) *etc*. and is not relevant to the choice of reflections for refinement. *R*-factors based on *F*^2^ are statistically about twice as large as those based on *F*, and *R*- factors based on ALL data will be even larger.

### Fractional atomic coordinates and isotropic or equivalent isotropic displacement parameters (Å^2^)

**Table:**

	*x*	*y*	*z*	*U*_iso_*/*U*_eq_	
Cd1	0.13088 (2)	0.599652 (19)	0.344239 (17)	0.05361 (14)	
N1	0.1810 (2)	0.5542 (2)	0.4686 (2)	0.0615 (10)	
N2	0.2592 (2)	0.6574 (2)	0.39215 (19)	0.0516 (9)	
O1	0.11652 (18)	0.67905 (18)	0.23068 (16)	0.0602 (8)	
O2	0.0871 (2)	0.74927 (19)	0.32546 (17)	0.0611 (8)	
O3	0.1387 (2)	0.4729 (2)	0.2927 (3)	0.0977 (12)	
O4	0.0538 (2)	0.41826 (19)	0.1917 (2)	0.0701 (10)	
O1W	0.00362 (18)	0.57274 (19)	0.35468 (17)	0.0611 (8)	
H1WA	−0.0326	0.6100	0.3365	0.073*	
H1WB	−0.0137	0.5234	0.3371	0.073*	
C1	0.0942 (3)	0.7474 (3)	0.2590 (3)	0.0527 (11)	
C2	0.0782 (3)	0.8273 (3)	0.2111 (2)	0.0537 (11)	
C3	0.1193 (3)	0.8370 (3)	0.1546 (3)	0.0677 (14)	
H3	0.1545	0.7937	0.1473	0.081*	
C4	0.1087 (4)	0.9095 (3)	0.1096 (3)	0.0825 (17)	
H4	0.1387	0.9161	0.0740	0.099*	
C5	0.0541 (4)	0.9725 (3)	0.1166 (3)	0.0813 (16)	
H5	0.0455	1.0205	0.0848	0.098*	
C6	0.0121 (3)	0.9632 (3)	0.1716 (3)	0.0685 (14)	
H6	−0.0245	1.0060	0.1769	0.082*	
C7	0.0232 (3)	0.8913 (2)	0.2195 (3)	0.0540 (11)	
C8	0.0959 (3)	0.4138 (3)	0.2577 (4)	0.0675 (15)	
C9	0.0975 (3)	0.3293 (3)	0.3005 (3)	0.0578 (12)	
C10	0.1638 (4)	0.3135 (4)	0.3615 (4)	0.0935 (19)	
H10	0.2047	0.3546	0.3747	0.112*	
C11	0.1687 (5)	0.2361 (5)	0.4026 (4)	0.114 (2)	
H11	0.2144	0.2245	0.4411	0.137*	
C12	0.1072 (5)	0.1780 (4)	0.3865 (4)	0.101 (2)	
H12	0.1096	0.1276	0.4151	0.121*	
C13	0.0428 (3)	0.1938 (3)	0.3290 (3)	0.0691 (14)	
H13	0.0007	0.1538	0.3194	0.083*	
C14	0.0357 (3)	0.2671 (2)	0.2827 (2)	0.0491 (10)	
C15	0.2960 (3)	0.7114 (3)	0.3534 (2)	0.0619 (12)	
H15	0.2725	0.7195	0.3023	0.074*	
C16	0.3658 (3)	0.7551 (3)	0.3848 (3)	0.0577 (12)	
H16	0.3882	0.7925	0.3556	0.069*	
C17	0.4026 (3)	0.7432 (3)	0.4598 (2)	0.0523 (11)	
C18	0.3652 (3)	0.6872 (3)	0.4998 (2)	0.0514 (11)	
H18	0.3882	0.6778	0.5508	0.062*	
C19	0.2946 (3)	0.6456 (2)	0.4655 (2)	0.0446 (10)	
C20	0.2522 (3)	0.5850 (2)	0.5076 (3)	0.0484 (11)	
C21	0.2842 (3)	0.5611 (3)	0.5819 (3)	0.0575 (12)	
H21	0.3336	0.5840	0.6080	0.069*	
C22	0.2441 (3)	0.5040 (3)	0.6179 (3)	0.0589 (12)	
C23	0.1719 (3)	0.4713 (3)	0.5762 (3)	0.0750 (15)	
H23	0.1435	0.4311	0.5974	0.090*	
C24	0.1423 (3)	0.4981 (3)	0.5035 (3)	0.0761 (15)	
H24	0.0927	0.4763	0.4766	0.091*	
C25	0.4800 (3)	0.7895 (3)	0.4962 (3)	0.0717 (14)	
H25A	0.4703	0.8509	0.4960	0.108*	
H25B	0.5196	0.7774	0.4685	0.108*	
H25C	0.4994	0.7698	0.5473	0.108*	
C26	0.2776 (3)	0.4777 (4)	0.6985 (3)	0.0843 (16)	
H26A	0.3136	0.4294	0.7006	0.126*	
H26B	0.2342	0.4612	0.7203	0.126*	
H26C	0.3066	0.5256	0.7263	0.126*	

### Atomic displacement parameters (Å^2^)

**Table:**

	*U*^11^	*U*^22^	*U*^33^	*U*^12^	*U*^13^	*U*^23^
Cd1	0.0639 (3)	0.0397 (2)	0.0639 (2)	−0.00834 (15)	0.02867 (18)	−0.00204 (16)
N1	0.068 (3)	0.050 (2)	0.073 (3)	−0.012 (2)	0.031 (2)	0.012 (2)
N2	0.062 (2)	0.044 (2)	0.057 (2)	−0.0098 (17)	0.0290 (18)	0.0006 (17)
O1	0.074 (2)	0.0420 (17)	0.073 (2)	0.0006 (15)	0.0356 (17)	−0.0078 (15)
O2	0.078 (2)	0.0476 (17)	0.068 (2)	0.0056 (15)	0.0378 (17)	0.0039 (15)
O3	0.087 (2)	0.070 (2)	0.150 (3)	−0.021 (2)	0.057 (2)	−0.040 (2)
O4	0.084 (3)	0.0377 (18)	0.106 (3)	−0.0016 (17)	0.058 (2)	−0.0019 (19)
O1W	0.065 (2)	0.0444 (16)	0.080 (2)	−0.0043 (15)	0.0306 (17)	−0.0008 (16)
C1	0.055 (3)	0.039 (2)	0.073 (3)	−0.0036 (19)	0.033 (2)	0.001 (2)
C2	0.063 (3)	0.042 (2)	0.065 (3)	−0.007 (2)	0.032 (2)	−0.002 (2)
C3	0.082 (4)	0.054 (3)	0.080 (3)	−0.004 (3)	0.045 (3)	0.003 (3)
C4	0.113 (5)	0.073 (4)	0.078 (4)	−0.014 (3)	0.054 (3)	0.009 (3)
C5	0.115 (5)	0.050 (3)	0.087 (4)	−0.005 (3)	0.040 (3)	0.017 (3)
C6	0.088 (4)	0.041 (3)	0.083 (3)	−0.002 (2)	0.035 (3)	0.008 (2)
C7	0.063 (3)	0.035 (2)	0.068 (3)	−0.005 (2)	0.025 (2)	0.000 (2)
C8	0.062 (3)	0.044 (3)	0.116 (5)	−0.009 (2)	0.057 (3)	−0.029 (3)
C9	0.058 (3)	0.053 (3)	0.069 (3)	0.005 (2)	0.027 (2)	−0.026 (2)
C10	0.067 (4)	0.093 (5)	0.117 (5)	0.003 (3)	0.017 (4)	−0.044 (4)
C11	0.116 (5)	0.116 (5)	0.093 (4)	0.045 (5)	−0.008 (4)	−0.020 (4)
C12	0.125 (6)	0.089 (5)	0.082 (4)	0.030 (4)	0.013 (4)	−0.001 (4)
C13	0.090 (4)	0.052 (3)	0.069 (3)	0.017 (3)	0.026 (3)	0.003 (3)
C14	0.061 (3)	0.035 (2)	0.058 (3)	0.0070 (19)	0.0269 (19)	−0.0053 (19)
C15	0.072 (3)	0.065 (3)	0.055 (3)	−0.009 (3)	0.029 (2)	0.012 (2)
C16	0.066 (3)	0.049 (3)	0.068 (3)	−0.010 (2)	0.037 (3)	0.008 (2)
C17	0.066 (3)	0.036 (2)	0.065 (3)	−0.003 (2)	0.036 (2)	−0.004 (2)
C18	0.062 (3)	0.047 (2)	0.052 (2)	−0.003 (2)	0.027 (2)	−0.003 (2)
C19	0.056 (3)	0.032 (2)	0.055 (3)	0.0018 (19)	0.031 (2)	−0.0006 (19)
C20	0.056 (3)	0.036 (2)	0.062 (3)	0.0020 (19)	0.032 (2)	0.001 (2)
C21	0.066 (3)	0.052 (3)	0.064 (3)	−0.001 (2)	0.034 (2)	0.008 (2)
C22	0.069 (3)	0.054 (3)	0.068 (3)	0.006 (2)	0.042 (3)	0.010 (2)
C23	0.083 (4)	0.068 (3)	0.087 (4)	−0.013 (3)	0.045 (3)	0.025 (3)
C24	0.068 (3)	0.070 (3)	0.096 (4)	−0.016 (3)	0.030 (3)	0.020 (3)
C25	0.077 (4)	0.068 (3)	0.077 (3)	−0.023 (3)	0.033 (3)	−0.007 (3)
C26	0.106 (4)	0.083 (4)	0.079 (4)	−0.003 (3)	0.050 (3)	0.022 (3)

### Geometric parameters (Å, °)

**Table:**

Cd1—O3	2.186 (4)	C10—H10	0.9300
Cd1—O1W	2.270 (3)	C11—C12	1.357 (9)
Cd1—N2	2.327 (3)	C11—H11	0.9300
Cd1—N1	2.329 (4)	C12—C13	1.342 (8)
Cd1—O1	2.369 (3)	C12—H12	0.9300
Cd1—O2	2.420 (3)	C13—C14	1.397 (6)
N1—C20	1.335 (6)	C13—H13	0.9300
N1—C24	1.343 (5)	C14—C14^i^	1.482 (8)
N2—C19	1.339 (5)	C15—C16	1.366 (6)
N2—C15	1.346 (5)	C15—H15	0.9300
O1—C1	1.272 (5)	C16—C17	1.371 (6)
O2—C1	1.252 (5)	C16—H16	0.9300
O3—C8	1.240 (6)	C17—C18	1.386 (5)
O4—C8	1.244 (6)	C17—C25	1.505 (6)
O1W—H1WA	0.8498	C18—C19	1.373 (6)
O1W—H1WB	0.8499	C18—H18	0.9300
C1—C2	1.495 (6)	C19—C20	1.506 (5)
C2—C3	1.395 (6)	C20—C21	1.382 (6)
C2—C7	1.397 (6)	C21—C22	1.380 (6)
C3—C4	1.372 (6)	C21—H21	0.9300
C3—H3	0.9300	C22—C23	1.375 (7)
C4—C5	1.374 (7)	C22—C26	1.499 (6)
C4—H4	0.9300	C23—C24	1.364 (7)
C5—C6	1.382 (7)	C23—H23	0.9300
C5—H5	0.9300	C24—H24	0.9300
C6—C7	1.394 (6)	C25—H25A	0.9600
C6—H6	0.9300	C25—H25B	0.9600
C7—C7^i^	1.524 (8)	C25—H25C	0.9600
C8—C9	1.515 (7)	C26—H26A	0.9600
C9—C10	1.400 (8)	C26—H26B	0.9600
C9—C14	1.403 (6)	C26—H26C	0.9600
C10—C11	1.400 (9)		
			
O3—Cd1—O1W	92.06 (12)	C9—C10—H10	119.9
O3—Cd1—N2	110.50 (13)	C12—C11—C10	120.3 (6)
O1W—Cd1—N2	150.70 (11)	C12—C11—H11	119.8
O3—Cd1—N1	96.06 (15)	C10—C11—H11	119.8
O1W—Cd1—N1	89.48 (12)	C13—C12—C11	119.4 (6)
N2—Cd1—N1	70.32 (12)	C13—C12—H12	120.3
O3—Cd1—O1	95.00 (13)	C11—C12—H12	120.3
O1W—Cd1—O1	106.00 (11)	C12—C13—C14	123.5 (6)
N2—Cd1—O1	90.88 (11)	C12—C13—H13	118.2
N1—Cd1—O1	160.61 (12)	C14—C13—H13	118.2
O3—Cd1—O2	147.01 (15)	C13—C14—C9	117.6 (4)
O1W—Cd1—O2	85.21 (10)	C13—C14—C14^i^	114.8 (4)
N2—Cd1—O2	85.37 (12)	C9—C14—C14^i^	127.5 (3)
N1—Cd1—O2	116.74 (12)	N2—C15—C16	123.9 (4)
O1—Cd1—O2	54.80 (9)	N2—C15—H15	118.0
C20—N1—C24	117.8 (4)	C16—C15—H15	118.0
C20—N1—Cd1	118.7 (3)	C15—C16—C17	119.3 (4)
C24—N1—Cd1	123.5 (4)	C15—C16—H16	120.4
C19—N2—C15	117.1 (4)	C17—C16—H16	120.4
C19—N2—Cd1	118.3 (2)	C16—C17—C18	117.0 (4)
C15—N2—Cd1	124.0 (3)	C16—C17—C25	120.9 (4)
C1—O1—Cd1	92.7 (2)	C18—C17—C25	122.1 (4)
C1—O2—Cd1	90.8 (2)	C19—C18—C17	121.2 (4)
C8—O3—Cd1	141.8 (3)	C19—C18—H18	119.4
Cd1—O1W—H1WA	117.7	C17—C18—H18	119.4
Cd1—O1W—H1WB	112.5	N2—C19—C18	121.5 (4)
H1WA—O1W—H1WB	107.7	N2—C19—C20	116.1 (4)
O2—C1—O1	121.7 (4)	C18—C19—C20	122.4 (4)
O2—C1—C2	120.2 (4)	N1—C20—C21	121.1 (4)
O1—C1—C2	118.1 (4)	N1—C20—C19	115.9 (4)
C3—C2—C7	118.8 (4)	C21—C20—C19	123.0 (4)
C3—C2—C1	118.0 (4)	C22—C21—C20	121.1 (5)
C7—C2—C1	123.1 (4)	C22—C21—H21	119.5
C4—C3—C2	121.1 (5)	C20—C21—H21	119.5
C4—C3—H3	119.5	C23—C22—C21	116.9 (4)
C2—C3—H3	119.5	C23—C22—C26	121.2 (4)
C3—C4—C5	120.7 (5)	C21—C22—C26	121.9 (5)
C3—C4—H4	119.7	C24—C23—C22	119.7 (4)
C5—C4—H4	119.7	C24—C23—H23	120.2
C4—C5—C6	119.0 (5)	C22—C23—H23	120.2
C4—C5—H5	120.5	N1—C24—C23	123.4 (5)
C6—C5—H5	120.5	N1—C24—H24	118.3
C5—C6—C7	121.6 (5)	C23—C24—H24	118.3
C5—C6—H6	119.2	C17—C25—H25A	109.5
C7—C6—H6	119.2	C17—C25—H25B	109.5
C6—C7—C2	118.8 (4)	H25A—C25—H25B	109.5
C6—C7—C7^i^	116.4 (3)	C17—C25—H25C	109.5
C2—C7—C7^i^	124.6 (3)	H25A—C25—H25C	109.5
O3—C8—O4	125.9 (6)	H25B—C25—H25C	109.5
O3—C8—C9	115.5 (6)	C22—C26—H26A	109.5
O4—C8—C9	118.7 (4)	C22—C26—H26B	109.5
C10—C9—C14	118.7 (5)	H26A—C26—H26B	109.5
C10—C9—C8	117.9 (5)	C22—C26—H26C	109.5
C14—C9—C8	123.5 (4)	H26A—C26—H26C	109.5
C11—C10—C9	120.3 (6)	H26B—C26—H26C	109.5
C11—C10—H10	119.9		
			
O3—Cd1—N1—C20	113.7 (3)	C1—C2—C7—C6	−179.5 (4)
O1W—Cd1—N1—C20	−154.3 (3)	C3—C2—C7—C7^i^	−177.3 (5)
N2—Cd1—N1—C20	4.0 (3)	C1—C2—C7—C7^i^	3.9 (8)
O1—Cd1—N1—C20	−10.8 (6)	Cd1—O3—C8—O4	69.7 (8)
O2—Cd1—N1—C20	−69.9 (3)	Cd1—O3—C8—C9	−111.2 (6)
O3—Cd1—N1—C24	−65.9 (4)	O3—C8—C9—C10	−21.7 (6)
O1W—Cd1—N1—C24	26.1 (4)	O4—C8—C9—C10	157.6 (4)
N2—Cd1—N1—C24	−175.6 (4)	O3—C8—C9—C14	157.5 (4)
O1—Cd1—N1—C24	169.6 (3)	O4—C8—C9—C14	−23.3 (6)
O2—Cd1—N1—C24	110.5 (4)	C14—C9—C10—C11	1.4 (7)
O3—Cd1—N2—C19	−95.8 (3)	C8—C9—C10—C11	−179.4 (5)
O1W—Cd1—N2—C19	42.3 (4)	C9—C10—C11—C12	−3.8 (10)
N1—Cd1—N2—C19	−6.6 (3)	C10—C11—C12—C13	2.4 (10)
O1—Cd1—N2—C19	168.5 (3)	C11—C12—C13—C14	1.3 (9)
O2—Cd1—N2—C19	114.0 (3)	C12—C13—C14—C9	−3.6 (7)
O3—Cd1—N2—C15	93.2 (4)	C12—C13—C14—C14^i^	173.3 (5)
O1W—Cd1—N2—C15	−128.7 (3)	C10—C9—C14—C13	2.1 (6)
N1—Cd1—N2—C15	−177.6 (4)	C8—C9—C14—C13	−177.0 (4)
O1—Cd1—N2—C15	−2.5 (3)	C10—C9—C14—C14^i^	−174.3 (5)
O2—Cd1—N2—C15	−57.1 (3)	C8—C9—C14—C14^i^	6.5 (7)
O3—Cd1—O1—C1	166.2 (3)	C19—N2—C15—C16	−1.0 (7)
O1W—Cd1—O1—C1	72.6 (3)	Cd1—N2—C15—C16	170.2 (4)
N2—Cd1—O1—C1	−83.1 (3)	N2—C15—C16—C17	1.1 (7)
N1—Cd1—O1—C1	−69.2 (4)	C15—C16—C17—C18	−0.7 (6)
O2—Cd1—O1—C1	0.6 (2)	C15—C16—C17—C25	179.5 (4)
O3—Cd1—O2—C1	−27.6 (4)	C16—C17—C18—C19	0.2 (6)
O1W—Cd1—O2—C1	−114.0 (3)	C25—C17—C18—C19	−180.0 (4)
N2—Cd1—O2—C1	93.8 (3)	C15—N2—C19—C18	0.4 (6)
N1—Cd1—O2—C1	159.0 (3)	Cd1—N2—C19—C18	−171.2 (3)
O1—Cd1—O2—C1	−0.6 (2)	C15—N2—C19—C20	179.9 (4)
O1W—Cd1—O3—C8	22.1 (7)	Cd1—N2—C19—C20	8.3 (4)
N2—Cd1—O3—C8	−177.0 (7)	C17—C18—C19—N2	−0.1 (6)
N1—Cd1—O3—C8	111.8 (7)	C17—C18—C19—C20	−179.5 (4)
O1—Cd1—O3—C8	−84.2 (7)	C24—N1—C20—C21	−1.3 (6)
O2—Cd1—O3—C8	−62.3 (8)	Cd1—N1—C20—C21	179.0 (3)
Cd1—O2—C1—O1	1.1 (4)	C24—N1—C20—C19	178.2 (4)
Cd1—O2—C1—C2	−177.5 (4)	Cd1—N1—C20—C19	−1.4 (5)
Cd1—O1—C1—O2	−1.1 (4)	N2—C19—C20—N1	−4.5 (5)
Cd1—O1—C1—C2	177.5 (3)	C18—C19—C20—N1	175.0 (4)
O2—C1—C2—C3	151.3 (4)	N2—C19—C20—C21	175.0 (4)
O1—C1—C2—C3	−27.4 (6)	C18—C19—C20—C21	−5.5 (6)
O2—C1—C2—C7	−29.9 (7)	N1—C20—C21—C22	0.8 (6)
O1—C1—C2—C7	151.5 (4)	C19—C20—C21—C22	−178.7 (4)
C7—C2—C3—C4	2.4 (8)	C20—C21—C22—C23	1.0 (7)
C1—C2—C3—C4	−178.7 (5)	C20—C21—C22—C26	−179.8 (4)
C2—C3—C4—C5	−3.3 (8)	C21—C22—C23—C24	−2.1 (7)
C3—C4—C5—C6	2.4 (9)	C26—C22—C23—C24	178.6 (5)
C4—C5—C6—C7	−0.7 (9)	C20—N1—C24—C23	0.1 (7)
C5—C6—C7—C2	−0.1 (8)	Cd1—N1—C24—C23	179.7 (4)
C5—C6—C7—C7^i^	176.7 (5)	C22—C23—C24—N1	1.7 (8)
C3—C2—C7—C6	−0.7 (7)		

Symmetry codes: (i) −*x*, *y*, −*z*+1/2.

### Hydrogen-bond geometry (Å, °)

**Table:**

*D*—H···*A*	*D*—H	H···*A*	*D*···*A*	*D*—H···*A*
O1W—H1WB···O4^i^	0.85	1.78	2.632 (4)	174
O1W—H1WA···O1^i^	0.85	1.95	2.782 (4)	164

Symmetry codes: (i) −*x*, *y*, −*z*+1/2.
